# Age, pelvic incidence, facet joint angle and pedicle-facet angle as correlative factors for isthmic spondylolisthesis: a retrospective case control study

**DOI:** 10.1186/s12891-023-06569-6

**Published:** 2023-06-17

**Authors:** Qian Kong, Bohan Wei, Shuoqun Niu, Jiawen Liao, Yuliang Zu, Tao Shan

**Affiliations:** 1 Department of Clinical Medicine, Qingdao Medical College, Qingdao University, Qingdao, 266071 Shandong People’s Republic of China; 2 Department of Radiology, Qilu Hospital of Shandong University (Qingdao), Qingdao, 266035 Shandong People’s Republic of China; 3 Human Morphology Laboratory, School of Basic Medicine, Qingdao University, Qingdao, 266071 Shandong People’s Republic of China

**Keywords:** Facet joint, Spinopelvic balance, Pelvic incidence, Spondylolisthesis, Computed tomography

## Abstract

**Background:**

Isthmic spondylolisthesis (IS) is a common clinical disease with a high incidence rate. However, most current researches explain the clear pathogenesis from a single perspective. The aim of our study was to explore the relationships between multiple parameters in patients and find the potential risk factors of this disease.

**Methods:**

Our study retrospectively included 115 patients who were diagnosed with isthmic spondylolisthesis and the same number of individuals without spondylolisthesis. The following parameters were measured or collected: age, pelvic incidence (PI), facet joint angle (FJA) and pedicle-facet angle (P-F angle). The radiographic files were imported into Mimics Medical 20.0 and all collected data were analyzed using SPSS, version 26.0, statistical software.

**Results:**

The age was higher in IS group than in control group. PI was also higher in the IS group (50.99 ± 7.67) than in the control group (43.77 ± 9.30) significantly (P = 0.009). There was significant difference in cranial and average FJA tropism in L3-L4 level (P = 0.002, P = 0.006, respectively) and in L4-L5 level (P < 0.001). P-F angle of L4-L5 level showed significantly larger in IS group than in control group (P = 0.007).The logistic regression analysis showed a larger age, a greater L3-L4 cranial FJA tropism, and a greater L4-L5 cranial FJA tropism were potential predictors of IS, with an OR of 1.07, 1.28, and 1.39 respectively. The thresholds of the predictors were 60 years, 5.67°, and 8.97° according to the ROC curve. The linear regression equation was established: degree of slippage (%) = 0.220*age − 0.327* L3-4 cranial FJA tropism − 0.346* L4-5 average FJA tropism (F = 3.460, P = 0.011, r = 0.659).

**Conclusions:**

Our study revealed that isthmic spondylolisthesis may be related to multiple factors rather than a single factor. Age, PI, PJA and P-F angle are potentially associated with spondylolisthesis.

## Background

Spondylolisthesis is a common pathological condition with an estimated 3-5% incidence rate [1, 2]. Isthmic spondylolisthesis (IS) is one of the main types of spondylolisthesis defined as the anterior translation of one vertebral body relative to the next caudal segment as a result of an abnormality of the pars interarticularis [3] that appears most commonly at the L5-S1 level, with a grade of slippage of I-IV. The hamstring tightness and lower back or buttock pain as well as disability such as intermittent claudication associated with IS [4, 5] cause a significant burden of patients’ quality of life. The related factors of IS are thought to be complex and multifactorial [6, 7], believed to involve both integral factors like sagittal spinopelvic alignments and partial elements like the morphology of lumbar facet joints [8, 9]. Some researchers reported an increase in pelvic incidence (PI) among patients with IS compared to healthy volunteers [10–12]. The facet joint of slippery segment appears to be structurally asymmetrical and have a more transverse orientation, that is to say an increase in difference between the left and right facet joint angles (FJA), and an increase in pedicle-facet angle (P-F angle) [9, 13].

However, published studies focus mostly on the respective relationship between IS and one certain factor while the relationship between multiple parameters involving several vertebral segments of IS patients has rarely been reported. What’s more, the few studies discussing the correlation between the parameters and the grade of slippage is controversial.

Most previous studies used 2D sagittal radiographs to measure the spinopelvic parameters such as PI in the standing position, which leads to difficulties to obtain the superposition of the two femoral heads in practice because of the rotation of the pelvic and nonvertical projection of the X-ray. As a result, some researchers pick the midpoint on the line connecting the centers of the femoral heads as the reference point to measure PI, causing the appearance of systematic error [14]. It is crucial for pre-and-post operative evaluation of IS patients to obtain accurate PI. Therefore, the CT scans is used for PI measurement in this study.

The aim of this study is to use 3D reconstructed models from CT images to evaluate PI and the morphological change of facet joint, as well as the relationships between multiple parameters in IS patients.

## Materials and methods

Our study was designed as a retrospective study for clinical research. With the approval of institutional review board, we included 115 patients who were diagnosed with isthmic spondylolisthesis in Qilu Hospital of Shandong University (Qingdao) from June 30th, 2017 to October 30th, 2021(85 Meyerding grade I patients, 30 Meyerding grade II patients). The diagnosis is made according to the imaging which indicates the cone moves forward. The Meyerding grade of the spondylolisthesis was defined based on the degree of slippage in the lumbar spine(grade 0, no slip; grade I, a 1–25% slip; grade II, a 25–50% slip; grade III, a 51–75% slip; and grade IV, a 75–100% slip) [5]. The same number of individuals without IS over the same period were considered as the control group.

The inclusion criteria were: (1)18 years of age or older. (PI increases during childhood and then remains unchanged throughout adolescence and adulthood [15]) (2) The imaging data and case notes of all study subjects were reserved completely. (3) The structures such as the femoral head, the sacrum endplate surface, and the facet joints can be clearly seen in the image.

The exclusion criteria were: (1) Congenital spinal deformities including congenital failure of formation or segmentation. (2) History of spinal and pelvic surgery. (3) Diagnosis of lumbar scoliosis, lumbar trauma or infection, lumbar facet joint infection or invasive tumour. (4) Other types of spondylolisthesis, such as degenerative, isthmic, traumatic, dysplastic, or pathologic spondylolisthesis (according to the Wiltse Classification).

Image data was exported as Digital Imaging and Communications in Medicine (DICOM) files (ABD = 1 mm) and the DICOM format files was imported into Mimics Medical 20.0 for measurement.

### Measurement of radiographic parameters

CT images of the subjects were saved in DICOM format. These DICOM files were imported into Mimics Medical 20.0 (Materialise NV, Belgium). All radiographic parameters were measured by four researchers using this software, and the average value was obtained for analysis. The radiographic parameters include:

#### Pelvic Incidence

**Fig. 1:**
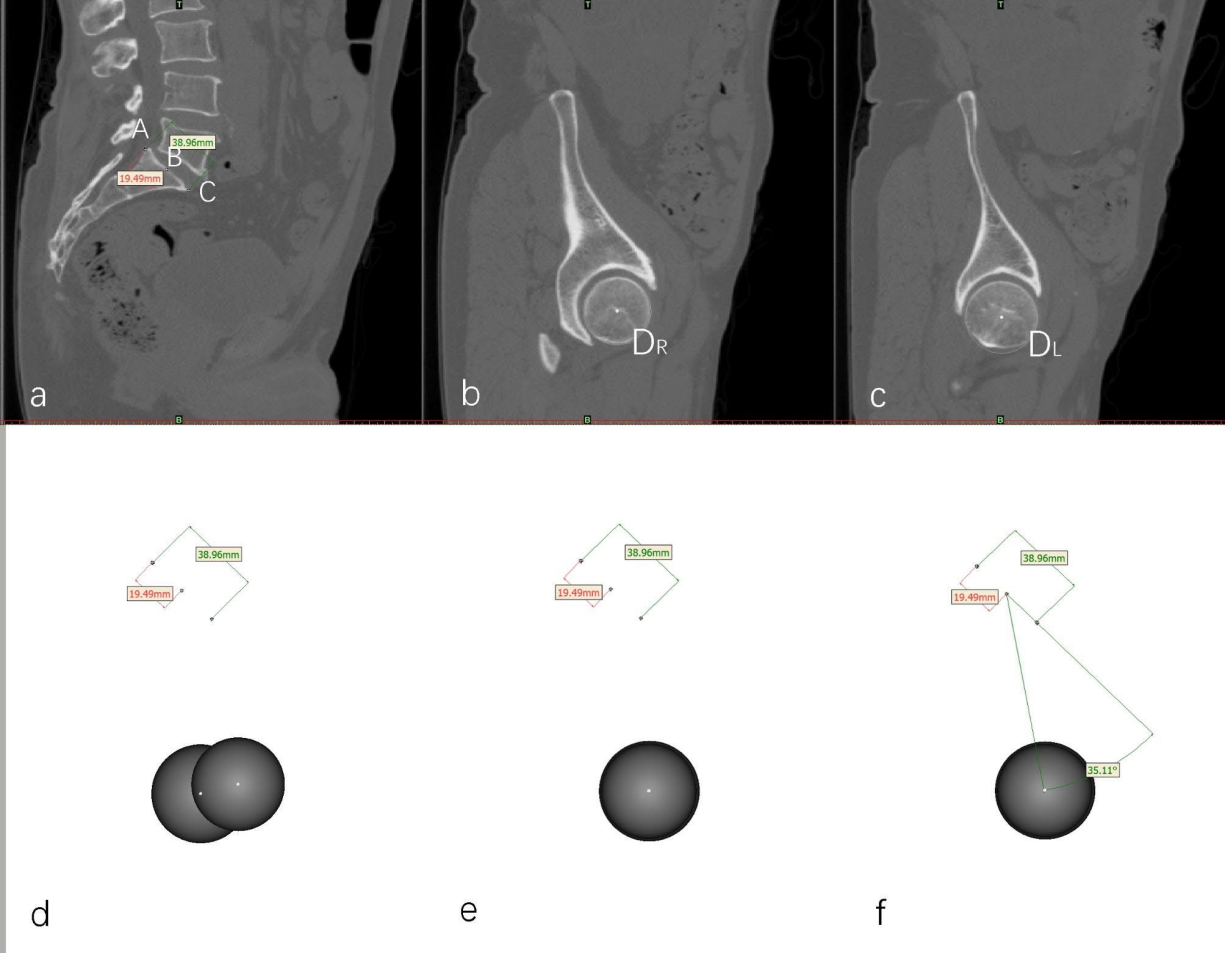
Measurement of pelvic incidence. **a** The posterior edge(A), midpoint(B) and anterior edge(C) of the sacral endplate are marked respectively in the sagittal view. **b-c** The approximate spheres of the left and right femoral heads are created using “sphere” tool, with which center of sphere(D_L_ and D_R_) could be automatically calculated. **d-e** In 3D view, the superposition of bilateral femoral heads was assured by rotating until the two centers coincide. **f** The angle between the line parallel to the sacral endplate and the line connecting the midpoint of sacral endplate to the center of femoral heads was measured, which is the complimentary angle of PI.

PI was measured using 3D reconstruction. We first marked the midpoint of sacral endplate using “point” tool, and created the approximate spheres of bilateral femoral heads using “sphere” tool in sagittal view. Then view was switched to 3D reconstruction to make sure the superposition of the left and right femoral heads. PI was measured by the complementary angle between the line parallel to the sacral endplate and the line connecting the midpoint of sacral endplate to the midpoint of the femoral heads [16] (Fig. 1).

#### Facet Joint Angle

**Fig. 2:**
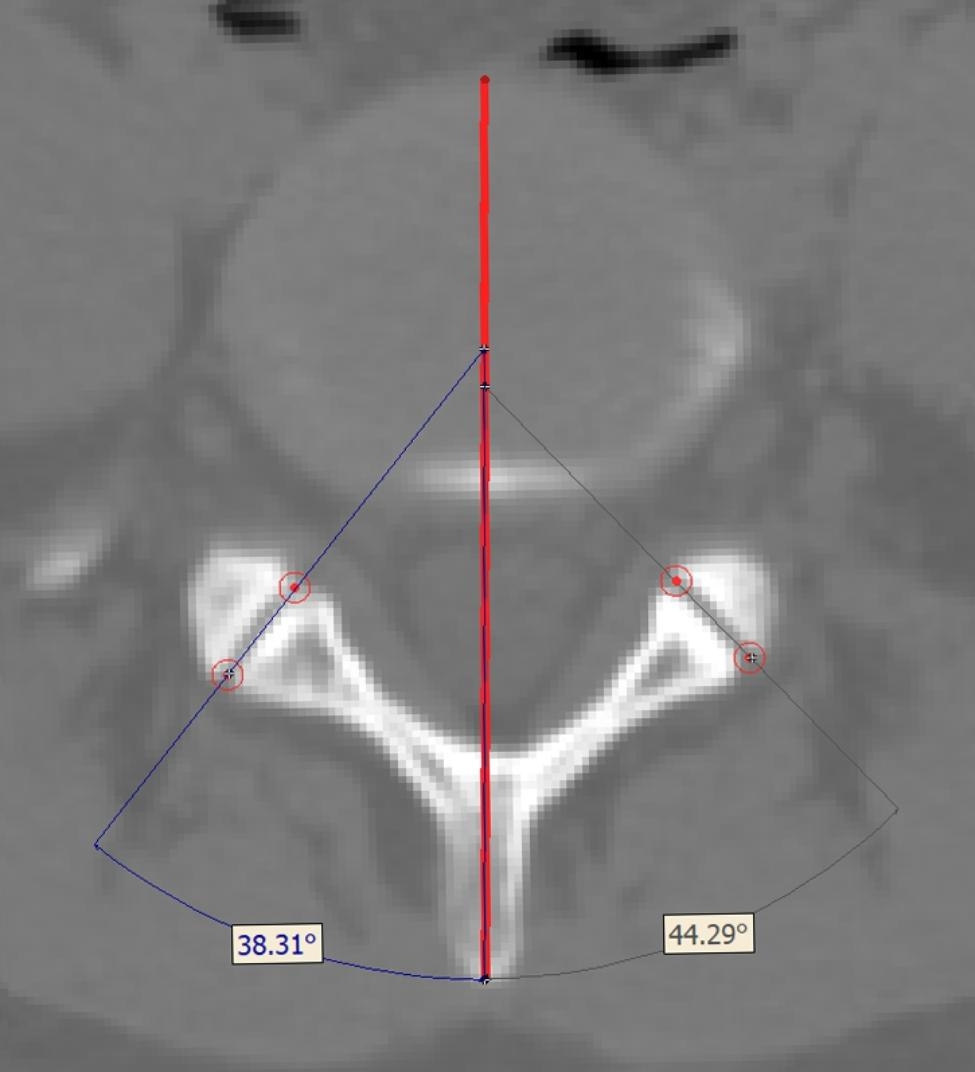
Measurement of facet joint angle. On an axial cut parallel to the vertebral endplate the FJA was measured. A line represents the sagittal axis of vertebra was drawn (the red line), then the anteromedial and posterolateral points of facet joint on both sides are marked. Connecting the anteromedial and posterolateral points, the angle between this line and the sagittal axis was measured as FJA.

FJA was measured on an axial cut parallel to the vertebral endplate. The angle was formed by a midsagittal line through the vertebral body and a line connecting the anteromedial point to the posterolateral point of the facet joint [17]. Both left and right side of the angles were measured (Fig. 2). The FJA measured in the plane that was parallel to endplate of the inferior vertebral body was defined as cranial FJA, while its counterpart measured in the plane that was parallel to endplate of the superior vertebral body was defined as caudal FJA. Both left and right, cranial and caudal FJA of L3-L4, L4-L5, and L5-S1 levels were measured.

#### Pedicle-facet Angle

**Fig. 3:**
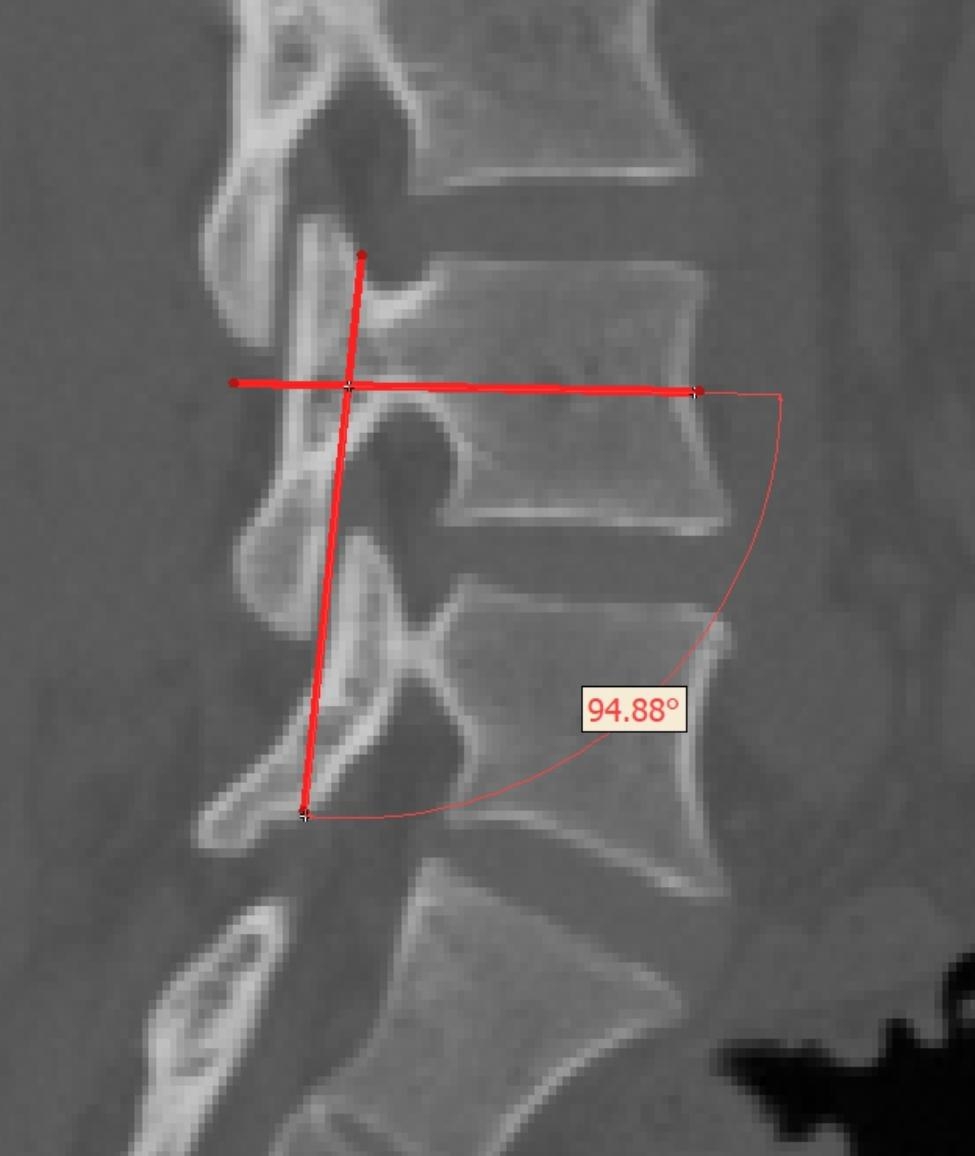
Measurement of pedicle-facet angle. In the sagittal view in which the facet joint was clearly visualized, the midpoints of anterior and posterior vertebral cortices were connected. The angle between this line and the line parallel to the facet joint was defined as P-F angle

P-F angle was measured on a sagittal cut in which the facet joint was clearly visualized. The angle between the line connecting the midpoint of the anterior and posterior vertebral body cortices and the line parallel to the facet joint was defined as P-F angle [18] (Fig. 3). P-F angle of L3-L4, L4-L5, and L5-S1 levels were measured.

#### Degree of slippage

The severity of isthmic spondylolisthesis can be described by degree of slip on the sagittal view. A line perpendicular to the upper endplate of the caudal vertebra was drawn from posterior border of the cranial vertebra. The distance posterior to the line divided by the width of caudal vertebra was defined as degree of slip [19].We measured the degree of slippage on L5 level and expressed them as a percentage.

### Statistical analysis

All collected data were analyzed using SPSS (version 26.0) and R (version 4.2.1), statistical software. Measures were expressed as the mean ± standard deviation. The between-group difference for PI, FJA and P-F angle of the spinal segments were analyzed using Student’s t-test. Pearson correlation analysis was performed in IS group to analyze the relationship between parameters and degree of slippage. Logistic regression analysis was used to analyze the potential predictors. Receiver operating characteristic curve (ROCC) and Youden index were used to analyze threshold for factors associated with lumbar IS. Linear regression analysis was performed to figure out the relationship between radiologic parameters and degree of slippage. Intraobserver and interobserver reliabilities were determined by intraclass correlation coefficient analysis. A probability (P) value of < 0.05 was considered statistically significant.

## Results

The IS group included 115 patients (53 male patients, 62 female patients) with an average age of 53.60 ± 16.24 years. We matched the same patients (53 male patients, 62 female patients) with an average of 62.25 ± 12.30 years. ICC analysis showed high intraobserver and interobserver reliabilities in the measurement of all parameters (0.855–0.959).

### Comparison of control and IS group

Age was higher in IS group than in control group (62.25 ± 12.46 vs. 53.60 ± 16.44, P = 0.010). PI was higher in the IS group than in the control group significantly (50.99 ± 7.67° vs. 43.77 ± 9.30°, P = 0.009). The FJA tropism shows different results in each segment. In L3-L4 level, there was significant difference in cranial and average FJA tropism (P = 0.002, P = 0.006, respectively). In L4-L5 level, there was also significant difference in cranial and average FJA tropism (P < 0.001). However, in L5-S1 level, no significant differences were observed in FJA tropism. P-F angle of L4-L5 level showed significantly larger in IS group than in control group (111.47 ± 7.57° vs. 106.74 ± 7.49°, P = 0.007) (Table 1).

**Table 1:** t-test of PI, FJA tropism, and P-F angle between IS and control group

			IS	control	t	P
Age			62.25 ± 12.46	53.60 ± 16.44	-2.652	**0.010**
PI			50.99 ± 7.67	43.77 ± 9.30	2.681	**0.009**
FJA tropism						
	L3-L4	cranial	9.80 ± 9.50	4.39 ± 4.08	3.286	**0.002**
		caudal	8.13 ± 8.26	5.53 ± 3.80	1.583	0.118
		average	8.82 ± 7.66	5.09 ± 3.05	2.855	**0.006**
	L4-L5	cranial	11.95 ± 7.35	5.10 ± 4.39	5.236	**0.000**
		caudal	7.90 ± 7.12	6.10 ± 5.54	2.310	0.194
		average	9.81 ± 6.01	5.49 ± 4.32	3.806	**0.000**
	L5-S1	cranial	9.26 ± 8.23	9.81 ± 7.23	-1.426	0.158
		caudal	7.58 ± 6.40	9.69 ± 6.70	-0.315	0.754
		average	8.40 ± 5.51	9.70 ± 5.83	-1.038	0.302
P-F angle						
	L3-L4		104.77 ± 5.75	104.39 ± 5.30	-0.303	0.763
	L4-L5		111.47 ± 7.57	106.74 ± 7.49	-2.772	**0.007**
	L5-S1		111.77 ± 7.28	110.60 ± 7.20	-0.671	0.505

The numbers in bold represented that there was significant difference between the two groups. FJA tropism was defined as the difference between left and right side on the same level (cranial or caudal). Average FJA tropism was defined as the average of cranial and caudal FJA tropism in a certain segment.

### Logistic regression analysis and threshold of the predictors

**Fig. 4:**
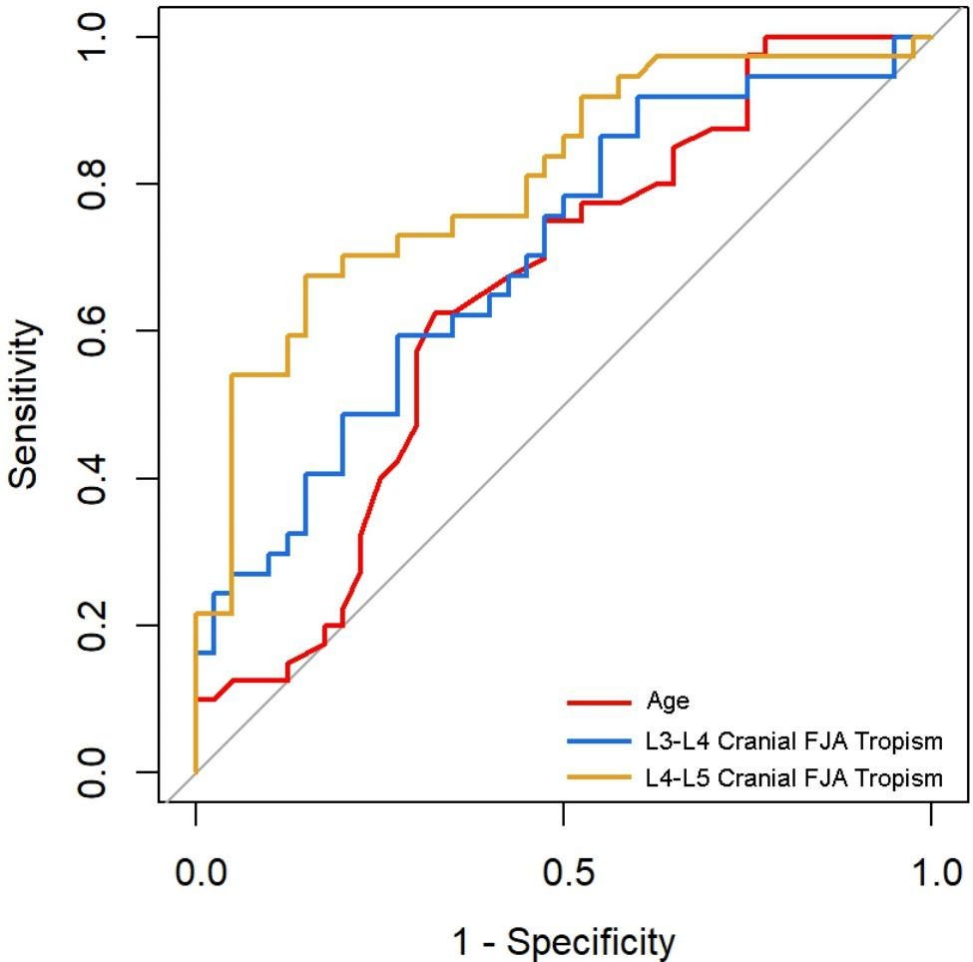
The ROC curve of predictors of IS. Age, L3-L4 cranial FJA tropism, and L4-L5 cranial FJA tropism were included in the ROC curve according to logistic regression analysis. The thresholds of the predictors were 60 years, 5.67°, and 8.97° respectively

Logistic regression analysis showed a larger age, a greater L3-L4 cranial FJA tropism, and a greater L4-L5 cranial FJA tropism were potential predictors of IS, with an OR of 1.07, 1.28, and 1.39 respectively (Table 2). The thresholds of the predictors were 60 years, 5.67°, and 8.97° according to the ROC curve (Fig. 4). See Table 3 for details.

**Table 2:** Logistic regression analysis of potential predictors

	β	OR	95% CI	P
Age	0.071	1.07	(1,1.15)	0.047
L3-L4 cranial FJA tropism	0.243	1.28	(1.06,1.54)	0.011
L4-L5 cranial FJA tropism	0.327	1.39	(1.13,1.7)	0.001

**Table 3:** Threshold of predictors of IS

	Sensitivity	Specificity	Youden Index	AUC	Threshold
Age	0.625	0.675	0.650	0.650	60.000
L3-L4 cranial FJA tropism	0.595	0.725	0.320	0.701	5.670
L4-L5 cranial FJA tropism	0.676	0.850	0.526	0.805	8.970

### Correlation between parameters and degree of slippage

Age was positively related with degree of slippage. There was a negative correlation between L3-L4 cranial FJA tropism and degree of slippage (r=-0.343, P = 0.024). A negative correlation could also be observed between L4-L5 average FJA tropism and degree of slippage (r=-0.318, P = 0.033). To further analyze the relationship between these parameters and degree of slippage, a linear regression equation was established as follows: degree of slippage (%) = 0.220*age − 0.327* L3-4 cranial FJA tropism − 0.346* L4-5 average FJA tropism (F = 3.460, P = 0.011, r = 0.659) (Table 4).

**Table 4:** Correlation coefficients between PI, FJA tropism, P-F angle and the degree of slippage in the 115 IS group

		r	P
Age		0.450	**0.007**
PI		-0.280	0.109
FJA tropism			
L3-L4	cranial	-0.343	**0.024**
	caudal	0.036	0.421
	average	-0.192	0.139
L4-L5	cranial	-0.145	0.068
	caudal	0.074	0.222
	average	-0.318	**0.033**
L5-S1	cranial	-0.001	0.497
	caudal	-0.004	0.481
	average	-0.003	0.487
P-F angle			
L3-L4		0.135	0.440
L4-L5		0.084	0.643
L5-S1		-0.040	0.841

## Discussion

In this study, we found the progression of IS may correlate with multiple factors, involving both integral factors like sagittal spinopelvic alignments and partial elements like the morphology of lumbar facet joints. Previous studies have mainly tended to be uni-factorial, with fewer studies looking at the factors associated with IS as a whole.

First, we found that FJA might have an impact on isthmic spondylolisthesis. In this study, the FJA of different vertebral segments were measured and the difference values were calculated. It was found that the L3-L4 cranial FJA tropism and the L4-L5 cranial FJA tropism in the spondylolisthesis group were significantly different from those in the control group, and the values of the relevant parameters in the spondylolisthesis group were higher than those in the control group. The risk of isthmic spondylolisthesis is possibly increased when the FJA tropisms were larger, especially if the L3-L4 cranial FJA tropism > 5.67, the L3-L4 cranial FJA tropism > 8.97 and age > 60 years. We proposed that the increase in FJA tropism may be related to the progression of IS. If excessive angle is repositioned below this threshold, it may improve the pelvic balance of the spine and provide guidance for clinical work.

In 1967, Farfan et al. proposed the concept of articular synovial asymmetry was that the synovial joint angle was increased on one side (tilted towards the coronal position) [20]. Ahmet Eroğlu et al. indicated that facet angle tropism could be seen in a high proportion of patients with IS. Masharaw YM also indicated that individuals with more frontally oriented facets in the lower lumbar vertebrae incorporated with facet tropism were at a greater risk for developing isthmic spondylolysis at L5 segment [21].

Further, the spondylolisthesis patients showed the significantly larger P-F angle of the L4-L5 segments and a larger PI than the normal group, which is consistent with the results of previous studies [22, 23]. T Iguchi and Fei Gao et al. certified the same conclusion that the P-F angle was significantly higher in the spondylolisthesis group than in the control group and it occurred more often in the L4-L5 segments [18, 24]. However, PI and P-F angle were only statistically significant in the Student’s t-test, but not in the logistic regression. The results may suggest that the PI and P-F angle have not directly impact the disease, but play an indirect indication of a tilted pelvis, resulting in a forward shift of the centre of gravity and a corresponding increase in the biological forces on the spine. It increases the isthmus shear forces and thus the risk of slipping. PI represents the anatomical position of the sacrum and the pelvis and has individual variability, so it becomes the reliable evaluation of the spinal-pelvic sagittal balance parameter. Previously, researchers believed that PI was the only anatomical and constant parameter while the others were positional parameters and remained constant in adults [25, 26]. However, the correlation between age and PI was observed significant in recent studies [27]. These findings might indicate that the occurrence of lumbar spondylolisthesis is not only correlated with a single related factor but a multifactorial interaction between related factors.

Our study shows that IS may be not merely related to the independent influence of a single relevant factor or the simple accumulation of multiple single factors. A possible process of how these parameters interact and contribute to lumbar spondylolisthesis is as follows. Patients with high PI have increased shear stresses at the lumbo-sacral junction, causing more tension on the pars interarticularis L5 segment, making the weak isthmus of the vertebral arch vulnerable to fatigue fractures [8, 28]. Similarly, Manon Sterba et al. [29] built a comprehensive osseo-disco-ligamentous L4–S1 finite element model to compute the Von Mises stress, L5–S1 facet contact force and resultant internal loads at the sacral endplate for each simulation and confirmed that the lumbosacral stresses increased with the higher PI, the posterior articular joint stresses of the vertebral body and the lumbosacral shear forces increased [30].

Meantime, the degeneration of the synovial joint probably increases after shear stress increasing in different lumbar segments, leading to the articular synovial asymmetry. One study has reported that disc degeneration predisposed to intersegmental instability and rotational strain, which resulted in secondary osteoarthritic change of the articular processes and segmental canal stenosis [31]. A possible explanation for this phenomena might be that symmetrica lumbar changes in the synovial joints may lead to disc disruption and reduced stability of the spine, while disruption of the disc may further cause an increase in intervertebral space-induced shear forces, resulting in isthmus fracture [32].

Li et al. measured the lamina angle on the L3 and L4 segments (the angle between the midline of the lower edge of the lamina and the posterior edge of the vertebral body). Both the lamina angle and the P-F angle reflect the degree of lamina tilt, and the values of the lamina angle were higher in the case group [32]. It is probably a result of the fact that the higher P-F angle, the greater inclination of the vertebral plate plane. The forces are more likely to be exerted at the level of the pedicle and the potential for fracture increases [33].

Our findings reveals that the IS is highly likely to be associated with a combination of factors. According to our study, the regression equation for slippage was obtained as: degree of slippage (%) = 0.220* age − 0.327* L3-L4 cranial FJA tropism − 0.346* L4-L5 average FJA tropism, which indicates that IS is not a disease just related with a single factor changes. It needs to be viewed from a multifactorial perspective. There was no significance in FJA between the two groups at L5-S1 segments, though all patients in this study had a slipped lumbar spine with an isthmic cleft at the L5 segments. Differences were found between the FJA in the L3-L4 and L4-L5 segments, and differences were found to be significant between the cranial FJA tropism in the L3-L4 and the L5-S1 segments. This suggests that parameters may interact with each other during the process of IS.

While the previous studies have reported differences in various spinal-pelvic parameters between IS and normal subjects, the disease progression of IS has rarely been analyzed from a holistic, multi-stage perspective. In our study, the ROCC showed that age, L3-L4 cranial FJA tropism, L4-L5 cranial FJA tropism were risk factors for IS, and the cut-off(threshold) values were obtained. The development of IS was investigated in terms of the integral factors of the spinal-pelvic sagittal balance, the local factors of the synovial joint angle and the P-F angle and the regression equation for the degree of slippage was further derived: degree of slippage (%) = 0.220* age − 0.327* L3-L4 cranial FJA tropism − 0.346* L4-L5 average FJA tropism, indicating that isthmic lumbar spondylolisthesis is a multifactorial disease process. This is where the present study differs from most studies. Based on the clinical reference to our results. Future studies further conduct relevant prospective trials to derive the causative relationship so that preventive treatment through intervention may be helpful to slow down the progression of IS or recover spinal-pelvic stability. Furthermore, the imaging technique of CT is used and has advantages in terms of the accuracy of the parameters results, which is different from the X-ray measurement method used in previous studies. Tomaz and Rob used CT scan methods to investigate the differences in PI between patients with lumbar scoliosis and normal subjects in order to provide increased reliability of the results [34, 35]. Another study compared PI obtained from lateral XR, standard CT scan and CT scan with 3D reconstruction and found PI measurements on repeated imaging of the same individual demonstrated that CT methods produced more consistent measurements than XR [36].

The present study has some limitations. First, this study is a retrospective study without including post-operative spinal-pelvic parameters to validate our structure, which might cause the bias. The study also needs to be tested in clinical practice to validate the results. Second, in order to get a more comprehensive understanding of the progression of IS, changes in micro-osseous structures [37] such as the pedicle and non-osseous structures[38] such as the intervertebral disc and ligaments should also be studied. Future retrospective analyses with larger sample sizes and multicentre large-scale randomized prospective clinical studies are awaited to verify the reliability of the results.

## Conclusion

IS is probably related to a combination of multiple factors. The age, L3-L4 cranial FJA tropism, L4-L5 cranial FJA tropism were possibly correlated with the process of isthmic lumbar spondylolisthesis and the regression equation was obtained as follows: degree of slippage (%) = 0.220* age − 0.327* L3-L4 cranial FJA tropism − 0.346* L4-L5 average FJA tropism. However, the study needs to be put into clinical practice to verify the results.

## Data Availability

The datasets used and/or analysed during the current study are available from the corresponding author on reasonable request.

## List of abbreviations

IS: Isthmic spondylolisthesis
CT: Computed tomography
PI: Pelvic incidence
FJA: Facet joint angle
P-F angle: Pedicle-facet angle
ROCC: Receiver Operating Characteristic Curve
XR: X-ray
OR: Odds ratio
95%CI: 95% Confidence interval
AUC: Area under the curve

## References

[1] Foreman P, Griessenauer CJ, Watanabe K, Conklin M, Shoja MM, Rozzelle CJ, Loukas M, Tubbs RS (2013). L5 spondylolysis/spondylolisthesis: a comprehensive review with an anatomic focus. Child’s Nerv system: ChNS : official J Int Soc Pediatr Neurosurg.

[2] Belfi LM, Ortiz AO, Katz DS (2006). Computed tomography evaluation of spondylolysis and spondylolisthesis in asymptomatic patients. Spine.

[3] Kreiner DS, Baisden J, Mazanec DJ, Patel RD, Bess RS, Burton D, Chutkan NB, Cohen BA, Crawford CH 3rd, Ghiselli G, et al. Guideline summary review: an evidence-based clinical guideline for the diagnosis and treatment of adult isthmic spondylolisthesis. The spine journal: official journal of the North American Spine Society. 2016;16(12):1478–85. 10.1016/j.spinee.2016.08.034 27592807

[4] Ren Z, Liu Y, Si X. The Effect Analysis of Surgical treatment about lumbar spondylolisthesis. Guide of China Medicine 2010, 8(22):56–7.

[5] Koslosky E, Gendelberg D. Classification in brief: the Meyerding classification system of Spondylolisthesis. Clin Orthop Relat Res 2020, 478(5):1125–30. 10.1097/CORR.0000000000001153 PMC7170696 32282463

[6] Jabłońska-Sudoł K, Maciejczak A. Relationship between the spino-pelvic parameters and the slip grade in isthmic spondylolisthesis. Neurologia i neurochirurgia polska 2015, 49(6):381–8. 10.1016/j.pjnns.2015.08.009 26652872

[7] Tebet MA (2014). Current concepts on the sagittal balance and classification of spondylolysis and spondylolisthesis. Revista brasileira de ortopedia.

[8] Roussouly P, Gollogly S, Berthonnaud E, Labelle H, Weidenbaum M. Sagittal alignment of the spine and pelvis in the presence of L5-s1 isthmic lysis and low-grade spondylolisthesis. Volume 31. Spine; 2006. pp. 2484–90. 21. 10.1097/01.brs.0000239155.37261.69 17023859

[9] Jiang H, Wang J, Yang X, Lai Z, Wu J, Wu F, Xian Z, Liu Z. Analysis of the related factors of lumbar spondylolysis. Chin J Clin Anat 2019, 37(05):583–5.

[10] Labelle H, Roussouly P, Berthonnaud E, Transfeldt E, O’Brien M, Chopin D, Hresko T, Dimnet J. Spondylolisthesis, pelvic incidence, and spinopelvic balance: a correlation study. Spine 2004, 29(18):2049–54. 10.1097/01.brs.0000138279.53439.cc 15371707

[11] Hanson DS, Bridwell KH, Rhee JM, Lenke LG. Correlation of pelvic incidence with low- and high-grade isthmic spondylolisthesis. Spine 2002, 27(18):2026–9. 10.1097/00007632-200209150-00011 12634563

[12] Labelle H, Roussouly P, Berthonnaud E, Dimnet J, O’Brien M (2005). The importance of spino-pelvic balance in L5-s1 developmental spondylolisthesis: a review of pertinent radiologic measurements. Spine.

[13] Eroğlu A, Çarlı BA, Pusat S, Şimşek H (2017). The role of the features of Facet Joint Angle in the development of Isthmic Spondylolisthesis in Young Male patients with L5-S1 isthmic spondylolisthesis. World Neurosurg.

[14] Lee KY, Lee JH, Im SK, Lee WY (2022). Analysis of measurement changes in pelvic incidence according to pelvic rotation using a three-dimensional model. BMC Musculoskelet Disord.

[15] Mangione P, Gomez D, Senegas J. Study of the course of the incidence angle during growth. European spine journal: official publication of the european spine Society, the european spinal deformity Society, and the european section of the cervical spine Research Society 1997, 6(3):163–7. 10.1007/BF01301430 PMC3454616 9258633

[16] Duval-Beaupère G, Schmidt C, Cosson P (1992). A barycentremetric study of the sagittal shape of spine and pelvis: the conditions required for an economic standing position. Ann Biomed Eng.

[17] Noren R, Trafimow J, Andersson GB, Huckman MS. The role of facet joint tropism and facet angle in disc degeneration. Spine 1991, 16(5):530–2. 10.1097/00007632-199105000-00008 2052995

[18] Gao F, Hou D, Zhao B, Sun X, Sun H, Li N, Guo L, Liu C (2012). The pedicle-facet angle and tropism in the sagittal plane in degenerative spondylolisthesis: a computed tomography study using multiplanar reformations techniques. J Spin Disord Tech.

[19] Cheung JPY, Fong HK, Cheung PWH. Predicting spondylolisthesis correction with prone traction radiographs. The bone & joint journal 2020, 102–b(8):1062–1071. 10.1302/0301-620X.102B8.BJJ-2020-0528.R1 32731831

[20] Farfan HF, Sullivan JD (1967). The relation of facet orientation to intervertebral disc failure. Can J Surg J canadien de chirurgie.

[21] Masharawi YM, Alperovitch-Najenson D, Steinberg N, Dar G, Peleg S, Rothschild B, Salame K, Hershkovitz I (2007). Lumbar facet orientation in spondylolysis: a skeletal study. Spine.

[22] Lv Z, Cao X, Guo Y, Ding J. Correlation analysis between spinal pelvic parameters and the degree of lumbar spondylolisthesis. Shandong Med J 2017, 57(48):54–6.

[23] Nie T, Xie S, Lv X, Lai Q, Dai M (2018). Relationship between spinal and pelvic sagittal parameters and degenerative lumbar spondylolisthesis. J Nanchang Univ (Medical Sciences).

[24] Iguchi T, Wakami T, Kurihara A, Kasahara K, Yoshiya S, Nishida K (2002). Lumbar multilevel degenerative spondylolisthesis: radiological evaluation and factors related to anterolisthesis and retrolisthesis. J Spin Disord Tech.

[25] Legaye J, Duval-Beaupère G, Hecquet J, Marty C. Pelvic incidence: a fundamental pelvic parameter for three-dimensional regulation of spinal sagittal curves. European spine journal: official publication of the european spine Society, the european spinal deformity Society, and the european section of the cervical spine Research Society 1998, 7(2):99–103. 10.1007/s005860050038 PMC3611230 9629932

[26] Schlösser TP, Janssen MM, Vrtovec T, Pernuš F, Oner FC, Viergever MA, Vincken KL, Castelein RM. Evolution of the ischio-iliac lordosis during natural growth and its relation with the pelvic incidence. European spine journal: official publication of the european spine Society, the european spinal deformity Society, and the european section of the cervical spine Research Society 2014, 23(7):1433–41. 10.1007/s00586-014-3358-z 24838427

[27] Jean L. Influence of age and sagittal balance of the spine on the value of the pelvic incidence. European spine journal: official publication of the european spine Society, the european spinal deformity Society, and the european section of the cervical spine Research Society 2014, 23(7):1394–9. 10.1007/s00586-014-3207-0 24509774

[28] Labelle H, Mac-Thiong JM, Roussouly P. Spino-pelvic sagittal balance of spondylolisthesis: a review and classification. European spine journal: official publication of the european spine Society, the european spinal deformity Society, and the european section of the cervical spine Research Society 2011, 20 Suppl 5(Suppl 5):641–6. 10.1007/s00586-011-1932-1 PMC3175928 21809015

[29] Sterba M, Arnoux PJ, Labelle H, Warner WC, Aubin C. Biomechanical analysis of spino-pelvic postural configurations in spondylolysis subjected to various sport-related dynamic loading conditions. European spine journal: official publication of the european spine Society, the european spinal deformity Society, and the european section of the cervical spine Research Society 2018, 27(8):2044–52. 10.1007/s00586-018-5667-0 29926211

[30] Li Y, Sun T, Ma B, Zhou Z, Dong R, Wu H (2022). A comparative study of imaging parameters and quality of life scores between subtypes of lumbar spondylolisthesis. Chin J Tissue Eng Res.

[31] Inoue S, Watanabe T, Goto S, Takahashi K, Takata K, Sho E (1988). Degenerative spondylolisthesis. Pathophysiology and results of anterior interbody fusion. Clin Orthop Relat Res.

[32] Li N, Zeng Y, Zhao C, Deng X, Wang Y (2021). Analysis of imaging factors related to isthmic spondylolisthesis. Chin J Spine Spinal Cord.

[33] Wang Z. Affection of pelvic anatomy and sagittal balance if the spine to isthmic spondylolisthesis etiology and clinical symptom. *MA thesis* Central South University; 2009.

[34] Vrtovec T, Janssen MM, Pernuš F, Castelein RM, Viergever MA (2012). Analysis of pelvic incidence from 3-dimensional images of a normal population. Spine.

[35] Brink RC, Vavruch L, Schlösser TPC, Abul-Kasim K, Ohlin A, Tropp H, Castelein RM, Vrtovec T. Three-dimensional pelvic incidence is much higher in (thoraco)lumbar scoliosis than in controls. European spine journal: official publication of the european spine Society, the european spinal deformity Society, and the european section of the cervical spine Research Society 2019, 28(3):544–50. 10.1007/s00586-018-5718-6 30128762

[36] Lee CM, Liu RW. Comparison of pelvic incidence measurement using lateral x-ray, standard ct versus ct with 3d reconstruction. European spine journal: official publication of the european spine Society, the european spinal deformity Society, and the european section of the cervical spine Research Society 2022, 31(2):241–7. 10.1007/s00586-021-07024-7 34743245

[37] Matthews PG, Phan K, Rao PJ, Ball JR (2015). Pedicle length and degree of slip in lumbosacral isthmic spondylolisthesis. Orthop Surg.

[38] Zhang K, Liu H, Wang J, Yu B, Chen T, Zheng Z, Huang Z (2015). The relationship between spinopelvic sagittal parameters and disc degeneration in patients with low-grade L5 isthmic spondylolisthesis. Orthop J China.

